# ECG signal classification in wearable devices based on compressed domain

**DOI:** 10.1371/journal.pone.0284008

**Published:** 2023-04-04

**Authors:** Jing Hua, Binbin Chu, Jiawen Zou, Jing Jia

**Affiliations:** 1 School of Software, Jiangxi Agricultural University, Nanchang, China; 2 School of Computer and Information Engineering, Jiangxi Agricultural University, Nanchang, China; Menoufia University, EGYPT

## Abstract

Wearable devices are often used to diagnose arrhythmia, but the electrocardiogram (ECG) monitoring process generates a large amount of data, which will affect the detection speed and accuracy. In order to solve this problem, many studies have applied deep compressed sensing (DCS) technology to ECG monitoring, which can under-sampling and reconstruct ECG signals, greatly optimizing the diagnosis process, but the reconstruction process is complex and expensive. In this paper, we propose an improved classification scheme for deep compressed sensing models. The framework is comprised of four modules: pre-processing; compression; and classification. Firstly, the normalized ECG signals are compressed adaptively in the three convolutional layers, and then the compressed data is directly put into the classification network to obtain the results of four kinds of ECG signals. We conducted our experiments on the MIT-BIH Arrhythmia Database and Ali Cloud Tianchi ECG signal Database to validate the robustness of our model, adopting Accuracy, Precision, Sensitivity and F1-score as the evaluation metrics. When the compression ratio (CR) is 0.2, our model has 98.16% accuracy, 98.28% average accuracy, 98.09% Sensitivity and 98.06% F1-score, all of which are better than other models.

## 1. Introduction

The World Health Organization’s 2022 report, Invisible numbers: The true extent of noncommunicable diseases and what to do about them, shows that deaths from non-communicable diseases account for three quarters of all deaths globally. Cardiovascular diseases account for 44% of all noncommunicable disease deaths and are the deadliest disease worldwide, accounting for one-third of all deaths. And 86% of cardiovascular diseases can be prevented and delayed by prevention, treatment and elimination of risk factors [1].

Intelligent health monitoring technology brought by the development of artificial intelligence can detect serious abnormalities without direct contact with patients. The technology can simultaneously monitor heart rate (HR), blood oxygen saturation (SpO2) and body temperature, and give warnings when abnormal [2, 3].The ECG signal is an electrical signal from the heart, usually represented by an electrocardiogram, that accurately reflects the basic pathology of the user, such as a fast, slow, irregular heartbeat and other arrhythmias, and plays a very important role in the detection of cardiovascular disease [4]. With the development of deep learning for ECG signal detection, most of the ECG signals tend to be detected by computers, which at this stage have achieved better accuracy than manual classification by experts [5]. In intensive care units, wearable devices, and some smart bracelets, it is possible to detect the user’s ECG in real time and give warnings in case of abnormalities [6, 7]. However, continuous health monitoring means that new data is constantly generated, placing certain requirements on the device’s data storage as well as its battery capacity [8]. Especially in wearable devices, the ECG detection program is required to have relatively high detection efficiency while taking up as little storage as possible, and to be able to transmit part of the ECG signal to the remote end quickly [9]. In order to meet these conditions, developers usually perform a series of simple pre-processing of the detected raw ECG signal to improve the detection accuracy and shorten the detection time and transmission time.

As a kind of long time frequency signal, the ECG signal generated in the acquisition process is often accompanied by noise artifacts, which will affect the accuracy of detection to some extent. In order to eliminate these effects, most studies on ECG signal detection classification will perform waveform transformation on ECG signals during preprocessing, such as fast Fourier transform (FFT), short-time Fourier transform (STFT), continuous wavelet transform (CWT), etc. [10–12]. In addition to these basic transformations, modifications could further improve the accuracy of heartbeat classification, Prakash et al. implement empirical mode decomposition based on Stockwell transform (ST), empirical wavelet transform (EWT) and Empirical Mode Decomposition (EMD) technology transforms or decomposes ECG signals [13–16]. Furthermore, the transformed ECG signals show additional characteristics, which should be further processed using ternary chess pattern (TCP) or homeomorphically irreducible tree (HIT) [17, 18]. In a recent study, the authors go beyond the transformation of signals only in the time or frequency domain and propose a transformation method for ECG signals that combines both time-domain and frequency-domain features [19]. The ECG signal also has its own unique characteristics, and in classifying ECG signals, researchers often look for individual heartbeat cycles within a long period of complete ECG signals. As the most unique waveform in the ECG signal, the QRS wave is generally used to determine the heartbeat cycle, and the accuracy of the localization of the QRS wave will largely affect the subsequent classification [20]. After Pahlm and Sornmo first proposed the QRS wave localization algorithm, including the current deep learning-based QRS wave localization algorithm, they are well able to accurately localize the QRS waves in ECG signals [21–24]. Besides, there is an adaptive sliding window positioning R-wave heartbeat cycle cutting method. A standard heartbeat cycle begins with a P-wave and ends with a T-wave, and the authors measured the time sum of each of these intervals, demonstrating that a heartbeat segment is at least 2s in length. So this method only needs to locate the most significant R peaks in the ECG, and can quickly fetch a complete heartbeat fragment, and the fragments taken out by this method are of the same length and can be directly input into the classification model. We also used this positioning cut method [4, 25].

After a simple pre-processing and cutting of the ECG signal, the classification process can begin. Due to the specificity of wearable devices, it is required that the model for classification occupies as little memory as possible. Traditional classifiers such as Support vector machine (SVM) and k-nearest neighbor (KNN) have been relatively saturated with classification results [26–28]. With the development of deep learning, many lightweight neural networks based on Deep neural network (DNN), Convolutional neural network (CNN) or Bi-directional LSTMs (Bi-LSTMs) are used for ECG signal classification. Instead of the traditional classifier [29–34]. Moreover, in order to combine heartbeat data and classification network characteristics, some studies customize different loss functions to achieve better classification results [35, 36]. Another way to significantly reduce the equipment space occupied by the classification system and at the same time speed up the data transmission and classification is to compress the ECG signal data. There are two main approaches to compressing ECG signals: the first one is based on compressed sensing (CS) by compressing the ECG signal and then reconstructing it, and the second one is to compress the ECG signal without reconstructing it [37, 38]. Both methods have been very useful in wearable devices, and we use the second method that processes the compressed data directly.

The organization of the research work is as follows: Section 2 presents a review related to ECG signal classification with the main contributions of this paper. Section 3 presents the material and the proposed methodology. Section 4 shows the results and analysis of the study. The research is summarized in the conclusion.

## 2. Literature review

The Compressed sensing (CS) model proposed by David L. Donoho in 2007 has greatly improved the signal processing technique by breaking the original Nyquist-Shannon sampling law and eliminating the need to guarantee that the sampling frequency must be greater than twice the maximum frequency [39]. Once the method was proposed, it was soon applied substantially in the fields of magnetic resonance imaging and image processing. The application of CS on ECG signals is also very effective. Due to the large amount of ECG signal data, the ECG signal can also be reconstructed after using CS compression, which speeds up the data transmission time while ensuring the basic characteristics of the ECG signal and does not affect the medical staff’s judgment of the pathology [40]. With the continuous development of deep learning, Bora et al. have combined CS with generators in neural networks to eliminate the limitation of signal sparsity and speed up the signal reconstruction to some extent, but the overall reconstruction still takes a relatively long time [41]. Wu et al. proposed a framework that combines generators and meta-learning to optimize the reconstruction process, which significantly speeds up the reconstruction of signals [42]. After the next, more and more studies are combining deep learning with CS, using neural networks to compress as well as reconstruct the process.

In wearable devices, the compression and classification of ECG signals is processed in two main modes.

The first one is compressed on the wearable device, then the compressed data is transmitted to the remote end for reconstruction and classification, and finally the detection results are transmitted to the device user. Hua et al. used a binary measurement matrix for compression, which can greatly reduce the energy consumption of sensor nodes, the BSBL algorithm with better recovery performance was used in the reconstruction process, and Deep Neural Networks (DNN) was used in the classification process, which had 94% accuracy when the data was less compressed. However, the classification accuracy is not ideal at high compression ratios and only classifies the ECG signals into abnormal and normal categories [37]. Zheng et al. used Singular Value Decomposition (SVD) to compress and reconstruct the ECG signal and compared it on SVM as well as CNN classifiers, with an average accuracy of 96% when the number of singular values was 3 in the case of classification into N, PVC, R, and L. However, the method cannot specify the compression ratio, and the There is still room to improve the accuracy at high compression ratios [43]. Fira et al. investigated the classification performance of several different dimensionality reduction techniques on ECG signals and Electroencephalogram (EEG) signals, incorporating Laplacian Eigenmaps (LE), Locality Preserving Projections (LPP), and CS. Meanwhile, the classification accuracy of SVM, KNN, decision tree and other classifiers is compared. Experiments show that CS is computationally cheaper and more effective compared to LE and LPP, while using SVM as well as KNN as classifier classifies the best results [44]. Another work by Fira builds on this by comparing in more detail the effects of different projection matrices and dictionaries in CS on classification results and by proposing two CS methods specifically for ECG signals, patient-specific classical compression perception without pre-processing before projection (PSCCS) and cardiac pattern compression perception involving ECG signal pre-processing and segmentation of the cardiac cycle (CPCS). Experiments have shown that if the ECG signal of the same patient for 24 hours needs to be detected in the ambulatory ECG. Then the PSCCS method can be chosen, and if the purpose is to identify and classify abnormalities in the ECG signal, the CPCS method with the presence of pre-processing as well as segmentation is more effective [45].

The second method is to classify compressed data directly on the wearable device. This processing method can significantly reduce the amount of data to be processed, reducing energy consumption and extending battery life while also speeding up recognition. Braun et al. skipped the costly reconstruction process in compression perception and compressed the MNIST database using a random orthogonal observation matrix and classified the compressed data. The error rate of the measured data at a compression rate of 0.4 differs from the error rate of the uncompressed data by only 0.22%, demonstrating the feasibility of compressed domain classification [46]. Hua et al. applied this feasibility to ECG signals, which were compressed using principal component analysis (PCA) after pre-processing and finally classified using SVM, yielding an average accuracy of 98.05% at a perceptual rate of 0.7 and an average accuracy of 98.58% for uncompressed data [38]. Another study improved on this by developing a new QRS detection algorithm capable of finding the location of QRS waves directly on the compressed ECG measurements and then classifying them using a Deep Boltzmann Machines (DBM). The experimental results show that at a compression rate of 0.4, the accuracy tested on the MIT-BIH database is 90.00% and 89.38% on its own database [47].

In addition to the above two common models, Yildirim et al. proposed a nonlinear compression structure based on a self-encoder (CAE), which can perform compression as well as decompression operations on ECG signals, and designed a Long short-term memory (LSTM) model to identify the compressed ECG signals, which can not only increase the classification efficiency, but also recover the compressed data when necessary [48].

### 2.1 Problem statement

In the existing literature, the compression and classification of ECG signals are usually carried out in two steps.

In the compression stage, the method based on compressed sensing is to use measurement matrix to compress signals. However, for long-term ECG signals, the design of measurement matrix is very difficult. It is difficult to find an appropriate measurement matrix to compress a series of ECG signals, and the compression time is long, which is not suitable for wearable devices requiring rapid recognition. Many studies have proposed using SVD, PCA, LEE and other methods to compress signals. These compression methods have good applicability, but SVD cannot accurately control the compression ratio of ECG signals, and PCA and LEE cannot retain the features of original signals well under the extremely low compression ratio.

In the classification stage, although simple classifiers such as SVM, KNN and MLP can quickly recognize ECG signals, the accuracy of classification results is low, and they are not suitable for wearable devices that need to accurately identify heart rate abnormalities. With the development of deep learning, many classification networks based on CNN and LSTM have shown good results in the recognition of ECG signals. However, due to the small volume of compressed ECG signals, many classification networks of larger magnitude will consume a lot of time in the work, which is not good for rapid recognition.

To sum up, a lightweight network capable of fast compression and accurate recognition should be equipped with the actual application environment of heart rate monitoring combined with wearable devices.

### 2.2 Major contributions

The purpose of this study is to develop a model for compressing and classifying ECG signals in wearable devices with the smallest possible storage space and high classification accuracy. The main studies are distributed in the processes of ECG signal pre-processing, heartbeat segment localization cutting, compression and classification. Our contributions are listed below:

- We propose a convolutional neural network-based compression method that replaces the traditional compression method and quickly and efficiently compresses the ECG signal while preserving its signal characteristics and greatly reducing the time required for classification.
- We propose a lightweight classification network structure based on deep learning, which has faster training speed and can effectively classify the compressed ECG signals.
- We conducted experiments on two different ECG signal databases to demonstrate the robustness of our model. The experimental results show that the compression method and classification method proposed in this paper have higher classification accuracy than those of the same type.

## 3. Materials and methods

This article does not include any studies involving humans or animals.

**Fig 1:**
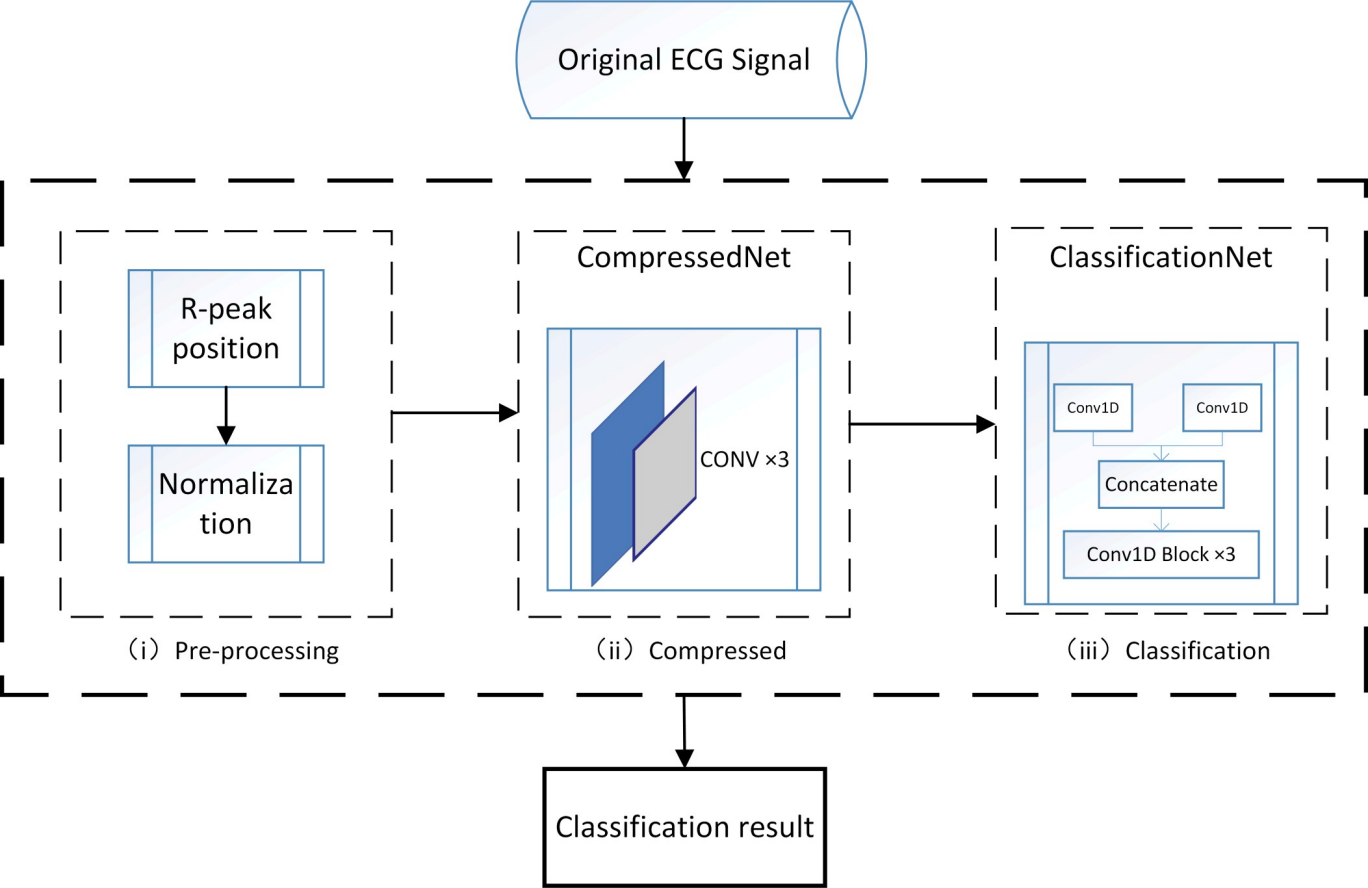
ECG signal compression classification process.

We propose a classification framework for compressed ECG signals, implementing an end-to-end ECG signal classification task that requires the input of the original ECG signal to derive its corresponding classification result, and the whole framework is shown in Fig 1. The raw ECG signal is first pre-processed, this stage normalizes and cuts each complete ECG signal into individual heartbeat beats, then these compressed using a compression network, and finally fed into a unique neural network for classification, resulting in classification results. The process of each step in the framework is described in detail below.

### 3.1 Datasets

**Fig 2:**
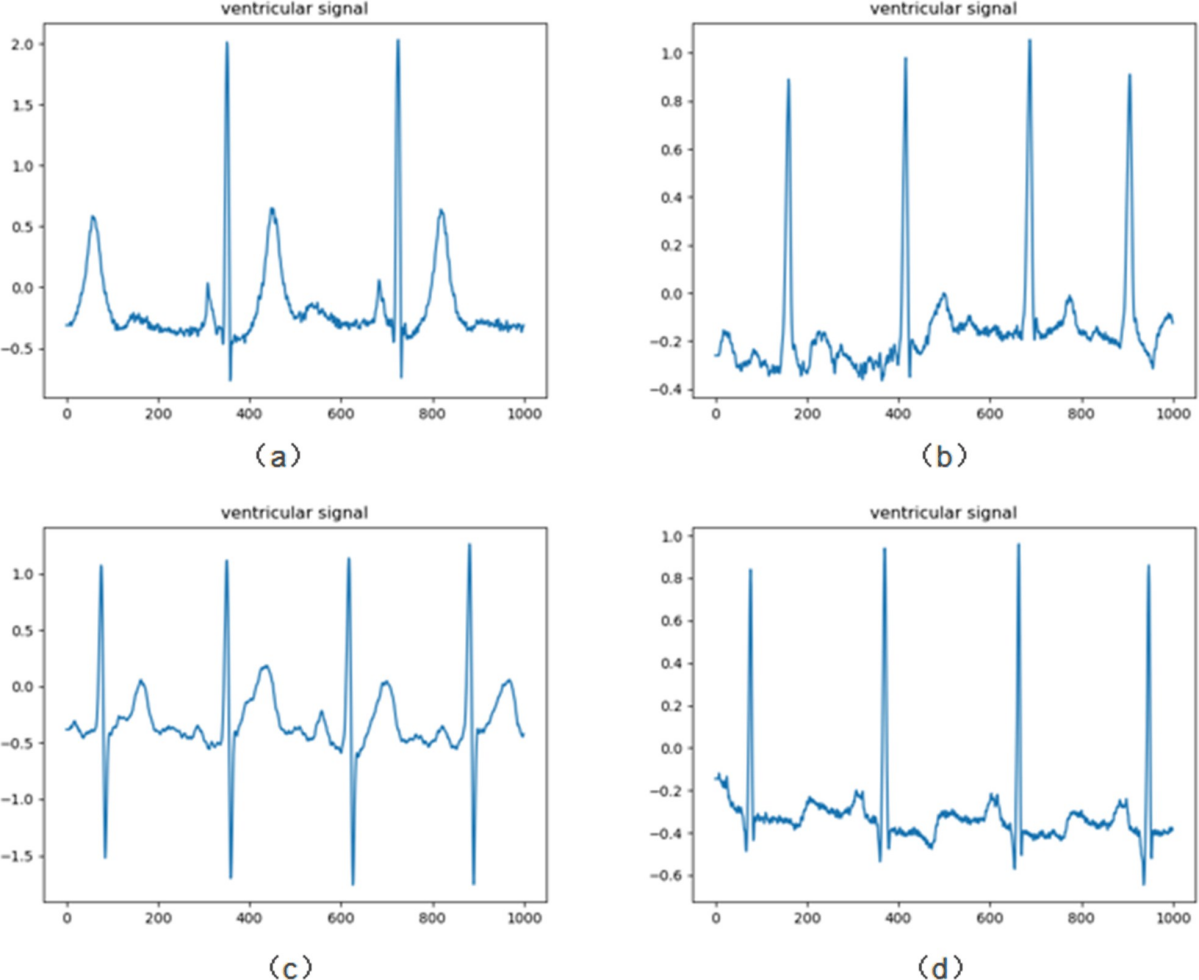
(a-d) ECG plotted for the first 1000 data of the subject.

We chose the MIT-BIH arrhythmia database provided by the Massachusetts Institute of Technology [49]. The database contains 48 dual-channel ambulatory ECG signal records from 47 subjects (records 201 and 202 are from the same subject), with the records digitized at a sampling rate of 360 samples per second per channel in the 10mV range at 11-bit resolution, and with each record annotated by two cardiologists separately. One of the ECG recordings consists of three parts: header file, data file and comment file. The header file records the file name, number of leads, sampling rate and number of data points, and the comment file records the diagnostic information of the ECG signal corresponding to the ECG expert. We selected all 48 signals in the MIT-BIH database and Fig 2 shows the ECG plotted for the first 1000 data of some subjects.

### 3.2 Pre-processing

In order to retain the original features of the ECG signal as much as possible and to enhance the generalization ability of the classification model, we did not filter the signal.

**Fig 3:**
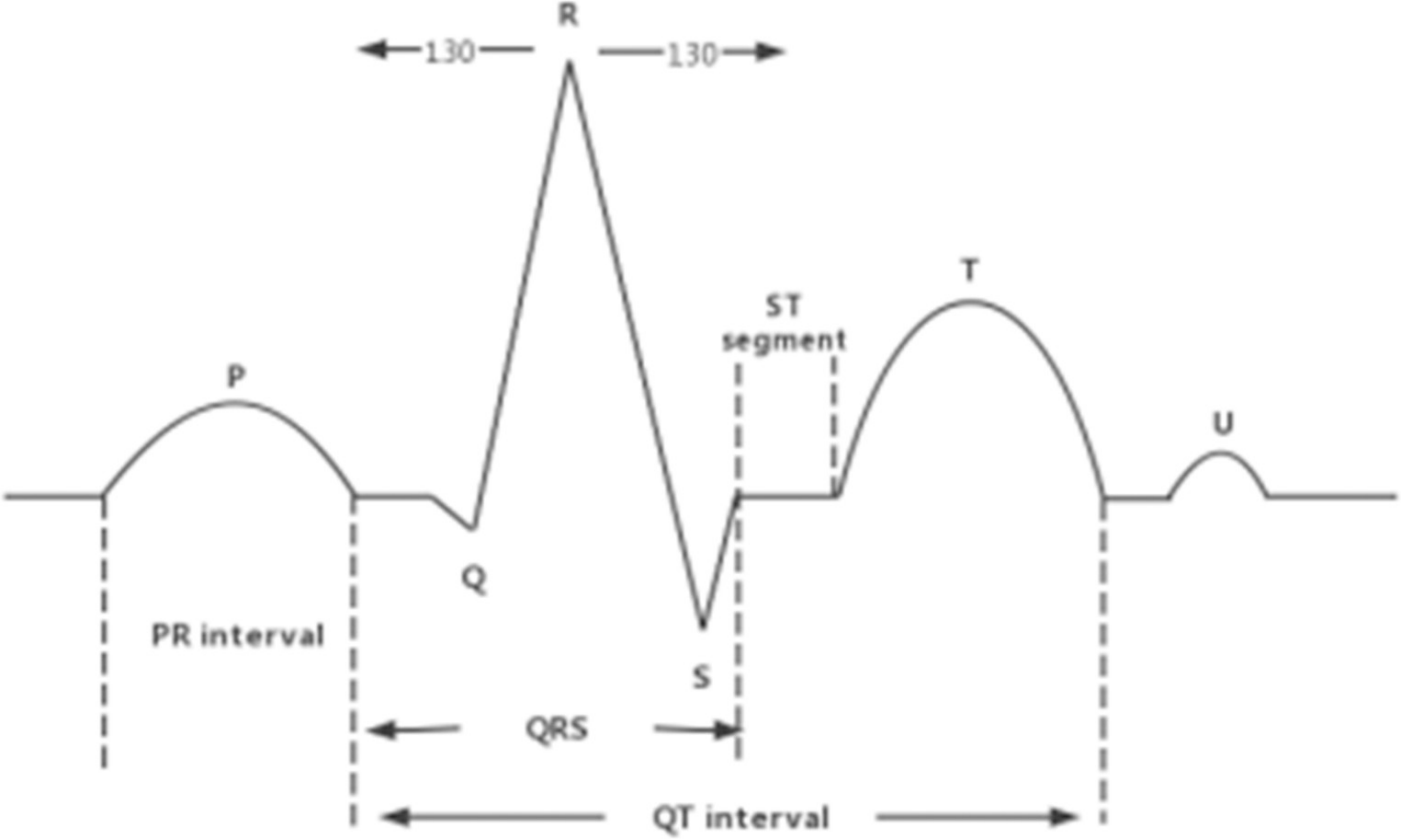
Schematic diagram of heartbeat beat interception centered on the R-peak.

To ensure better processing of the data by the classification model, the ECG signal is segmented by heartbeat beats before the data is fed into the model. A complete heartbeat beat is defined as the beginning of a P-wave to the end of a U-wave. As shown in Fig 3, the easiest way to intercept a heartbeat signal is to first locate the highest point, R, and then take 130 data on the left and right side, using R as the starting point, to form a heartbeat beat of 260 samples in length. Each beat has its own label category, and this heartbeat beat interception method can retain most of the feature information for classification.

All 48 ECG signals in the MIT-BIH arrhythmia database were segmented, resulting in a total of 109,451 heartbeat beats. The classification is based on the five ECG signals recommended by the AAMI standard, which are divided into five categories: Normal beat (N), Premature or ectopic supraventricular beat (S), Premature ventricular contraction (V), Fusion of ventricular and normal beat (F), and Unclassifiable beat (Q). The number of each of these categories is shown in Table 1. It can be seen that the number of heartbeats in category N is high, accounting for about 90.09% of the total number of categories, showing an imbalance in the data. If these heartbeat segments are directly fed into the classification model for training, it will lead to the inability of the classifier to perform accurate recognition. The classifier can obtain relatively high classification accuracy without learning even if it only classifies heartbeats into N classes, which results in unreliable data results. To avoid this data imbalance, some studies use GAN networks to generate categories with less data or to augment the dataset [50, 51]. Instead, we manually selected 1000 data for each of the N, V, and S heartbeat categories, 802 data for the F category, and discarded the Q heartbeats to form a new dataset with a total of 3802 heartbeat beats, which facilitates our training of the network model.

**Table 1:** Number of each heartbeat beat category after cutting and the actual number used [49].

AAMI category	MIT-BIH category	Count	Actual use
N	Normal beat	98600	1000
	Fusion of paced and normal beat		
	Atrial escape beat		
	Paced beat		
	Nodal (junctional) escape beat		
	Supraventricular escape beat		
	Left or right bundle branch block		
	Left bundle branch block beat		
	Right bundle branch block beat		
S	Premature or ectopic supraventricular beat	2781	1000
	Atrial premature contraction		
	Nodal (junctional) premature beat		
	Aberrated atrial premature beat		
V	Premature ventricular contraction	7235	1000
	Ventricular escape beat		
	R-on-T premature ventricular contraction		
F	Fusion of ventricular and normal beat	802	802
Q	Unclassifiable beat	33	-

### 3.3 The proposed compression method

Compressing ECG data can eliminate a large amount of redundant data and retain only the critical data that can be used for diagnosis. We define the compression ratio (CR) as:

$$CR=\frac{k}{n}$$ (1)

where n = 260 is the original data length and k is the compressed heartbeat beat length. We use compression rates of 0.5, 0.4, 0.3, 0.2, 0.1, and 0.05. Our proposed method is described below.

**Fig 4:**
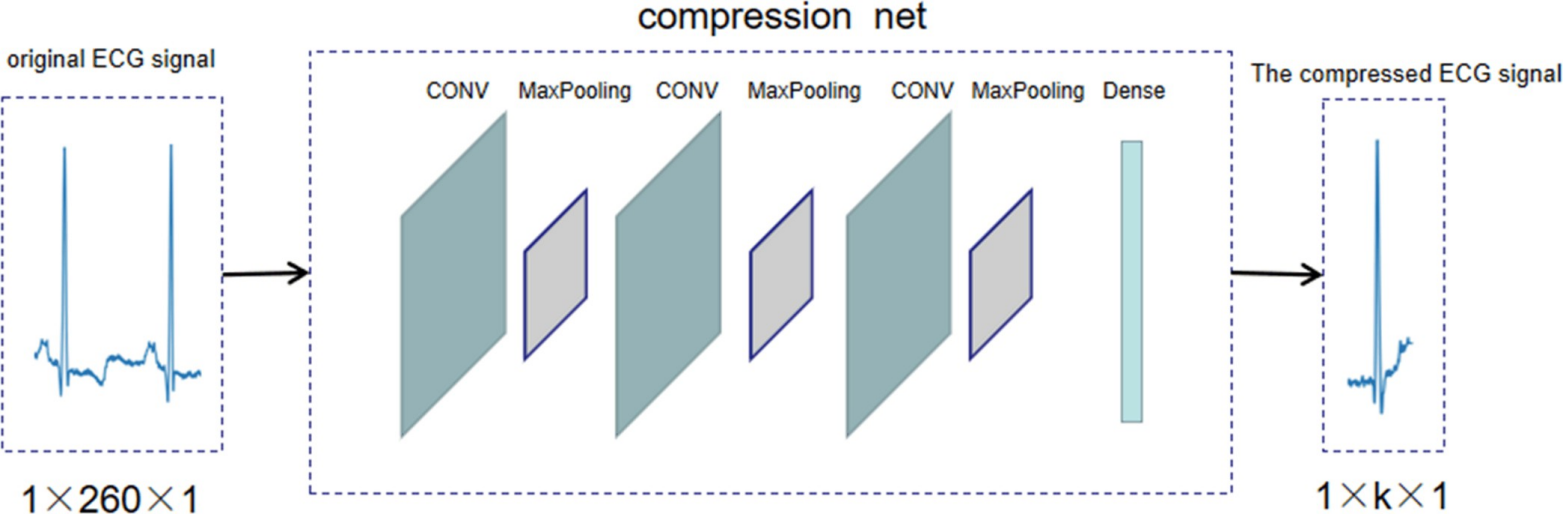
Proposed compression process.

We used a neural network instead of the traditional compression method, and the compression process Fig 4 is shown. After normalizing the data, the ECG signal is compressed by the compression network and a compressed ECG signal of a specified size is output. The compression network consists of three convolutional layers alternating with a maximum pooling layer, followed by a fully connected layer for specifying the compression dimension. Among them, the combination of convolutional and pooling layers can enhance and extract data features of ECG signals, reduce information redundancy, and prevent overfitting. Table 2 shows the detailed parameters of the compressed network at CR = 0.2. The amount of compressed data for an ECG signal of 260 is only 52 after the compressed network and can be used directly for the subsequent classification work.

**Table 2:** Detailed parameters of the compression network at CR = 0.2.

Layer	type	Convolution kernel number/Unit number	Convolution kernel size/step size	Output	Param #
1	**Input**	**-**	**-**	**260×1**	**0**
2	**Conv1D**	**16**	**3, 1**	**260×16**	**64**
3	**Maxpooling1D**	**-**	**2, 1**	**130×16**	**0**
4	**Conv1D**	**16**	**3, 1**	**130×16**	**784**
5	**Maxpooling1D**	**-**	**2, 1**	**65×16**	**0**
6	**Conv1D**	**16**	**3, 1**	**65×16**	**784**
7	**Maxpooling1D**	**-**	**5, 1**	**13×16**	**0**
8	**Dense**	**52**	**-**	**52**	**10868**

### 3.4 The proposed classification method

**Fig 5:**
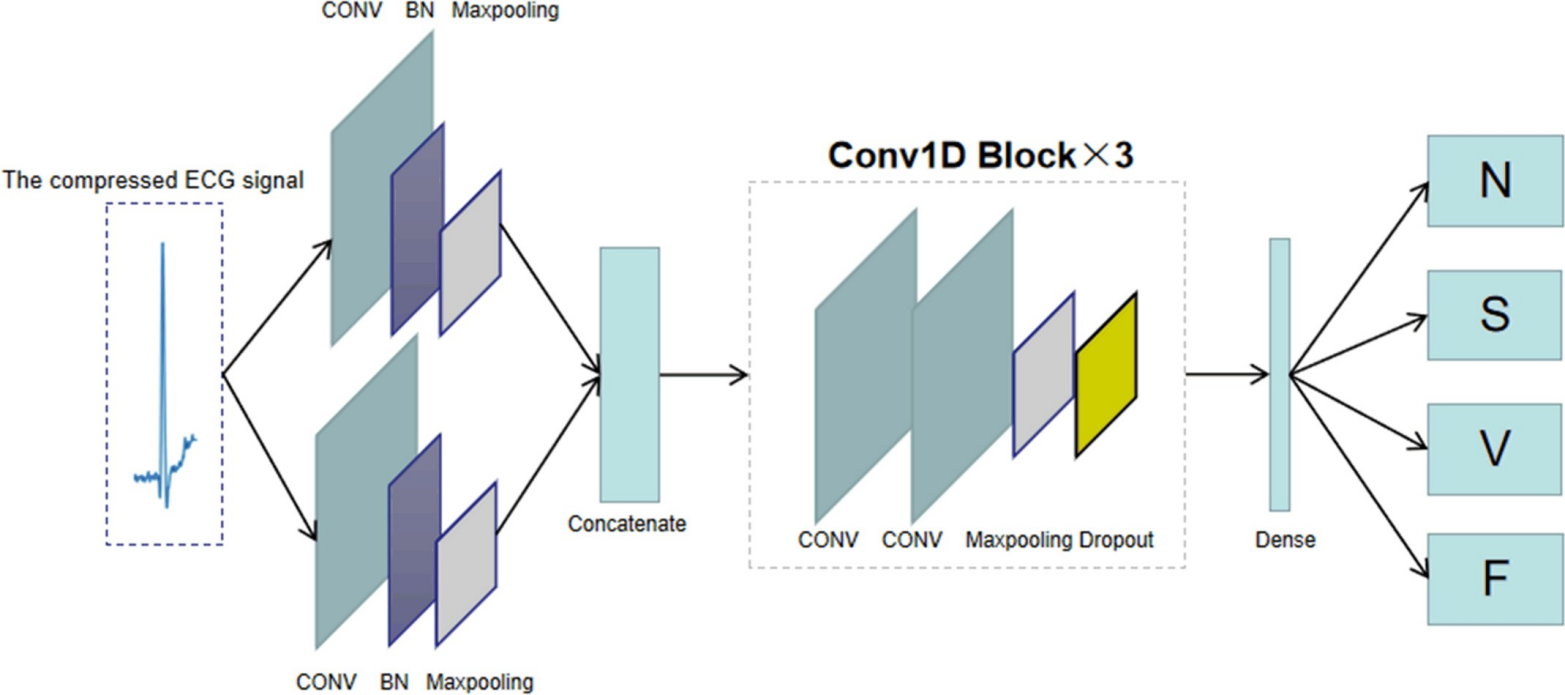
Proposed classification network.

To improve the accuracy of recognition of compressed data, we propose a classification network model for compressed ECG signals, as shown in Fig 5. The compressed ECG signal is fed into the classification network. Since the signal at low compression ratio contains fewer features, the network first processes the signal in parallel to better extract more features. Each parallel route is further extracted by a convolutional layer, followed by a BatchNormalization (BN) layer for normalization, which speeds up the convergence of the network while also preventing overfitting. Afterwards, the two parallel features are fused and fed into a structure consisting of three one-dimensional convolutional blocks. Finally, the softmax activation function of the fully connected layer is used for classification, resulting in four classification results. Table 3 shows the detailed parameters of the classification network at CR = 0.2, and some of the important structures in the network are described below.

**Table 3:** Detailed parameters of the classification network at CR = 0.2.

Layer	type	Convolution kernel number/Unit number	Convolution kernel size/step size	Output	Param #	Connected to
**1**	**Input**	**-**	**-**	**52×1**	**0**	**-**
**2~3**	**Conv1D**	**16**	**3, 1**	**52×16**	**64**	**1**
**4~5**	**BatchNormalization**	**-**	**-**	**52×16**	**64**	**2~3**
**6~7**	**Maxpooling1D**	**-**	**2, 1**	**26×16**	**0**	**4~5**
**8**	**Concatenate**	**-**	**-**	**52×16**	**0**	**6 and 7**
**9~10**	**Conv1D**	**16**	**3, 1**	**52×16**	**784**	**8**
**11**	**Maxpooling1D**	**-**	**2, 1**	**26×16**	**0**	**10**
**12**	**Dropout (0.5)**	**-**	**-**	**26×16**	**0**	**11**
**13**	**Conv1D**	**64**	**3, 1**	**26×64**	**3136**	**12**
**14**	**Conv1D**	**64**	**3, 1**	**26×64**	**12352**	**13**
**15**	**Maxpooling1D**	**-**	**3, 1**	**8×64**	**0**	**14**
**16**	**Dropout (0.5)**	**-**	**-**	**8×64**	**0**	**15**
**17~18**	**Conv1D**	**64**	**3, 1**	**8×64**	**12352**	**16**
**19**	**Maxpooling1D**	**-**	**4, 1**	**2×64**	**0**	**18**
**20**	**Dropout (0.5)**	**-**	**-**	**2×64**	**0**	**19**
**21**	**Flatten**	**-**	**-**	**128**	**0**	**20**
**22**	**Dense**	**4**	**-**	**4**	**516**	**21**

#### 3.4.1 1D-CNN

1D-CNN is mainly used for feature extraction of time-series data. The input shape of the network is (n, m), where n means there are n consecutive time-series data and m is the feature of each time-series data, which is taken as 1. The convolution kernel of 1D convolution needs to specify only one dimension, and the depth of the convolution kernel is automatically consistent according to the input layer depth. The essence of convolution is the multiplication and addition operation. Assume that the weight of each position of the convolution kernel is *W_ij_*, the value of each position of the corresponding convolution region is *x_ij_*, the output value of each convolution region after convolution is y, and b is the offset. The one-dimensional convolution is calculated as follows:

$$y=\sum_{i=1}^{k}\sum_{j=1}^{m}w_{ij}x_{ij}+b$$ (2)

#### 3.4.2 Concatenate

The Concatenate layer allows the fusion of multiple features, mainly for the merging of the number of channels, increasing the features described by the data without increasing the information under each feature. The Concatenate layer is able to fuse multiple convolutional features for ECG data with high compression ratios, increasing the number of features for different categories of ECG signals as much as possible, which facilitates the subsequent classification process.

## 4. Results and discussion

We performed network training and testing on Keras, a deep learning framework backed by Tensorflow, on a computer with an Intel(R) Xeon(R) Silver 4112 CPU @ 2.60GHz and an NVIDIA Quadro RTX5000 graphics card.

There are two major limitations in this study that could be addressed in future research. The first is the limitation of samples. In most ECG signal data sets, the number of abnormal ECG data is usually small. If these data are trained directly, the classifier will not be able to effectively identify abnormal ECG data. Although this study balanced the data set and ensured the training of the model, the ECG signal data of class F was still small, which affected the classification results to a certain extent. The method of balancing data sets will be further studied in the future. The second is the limitation of the method. The compressed domain classification method can improve the speed of ECG signal recognition. However, under normal circumstances, it is not guaranteed that CR is proportional to the classification accuracy. Only when the difference of CR is large, for example, CR = 0.5 is compared with 0.3, the accuracy rate of CR = 0.5 is higher. Moreover, due to the limitation of the length of ECG signal, the ECG signal compressed below CR = 0.05 cannot be effectively recognized.

### 4.1 Training parameters

**Fig 6:**
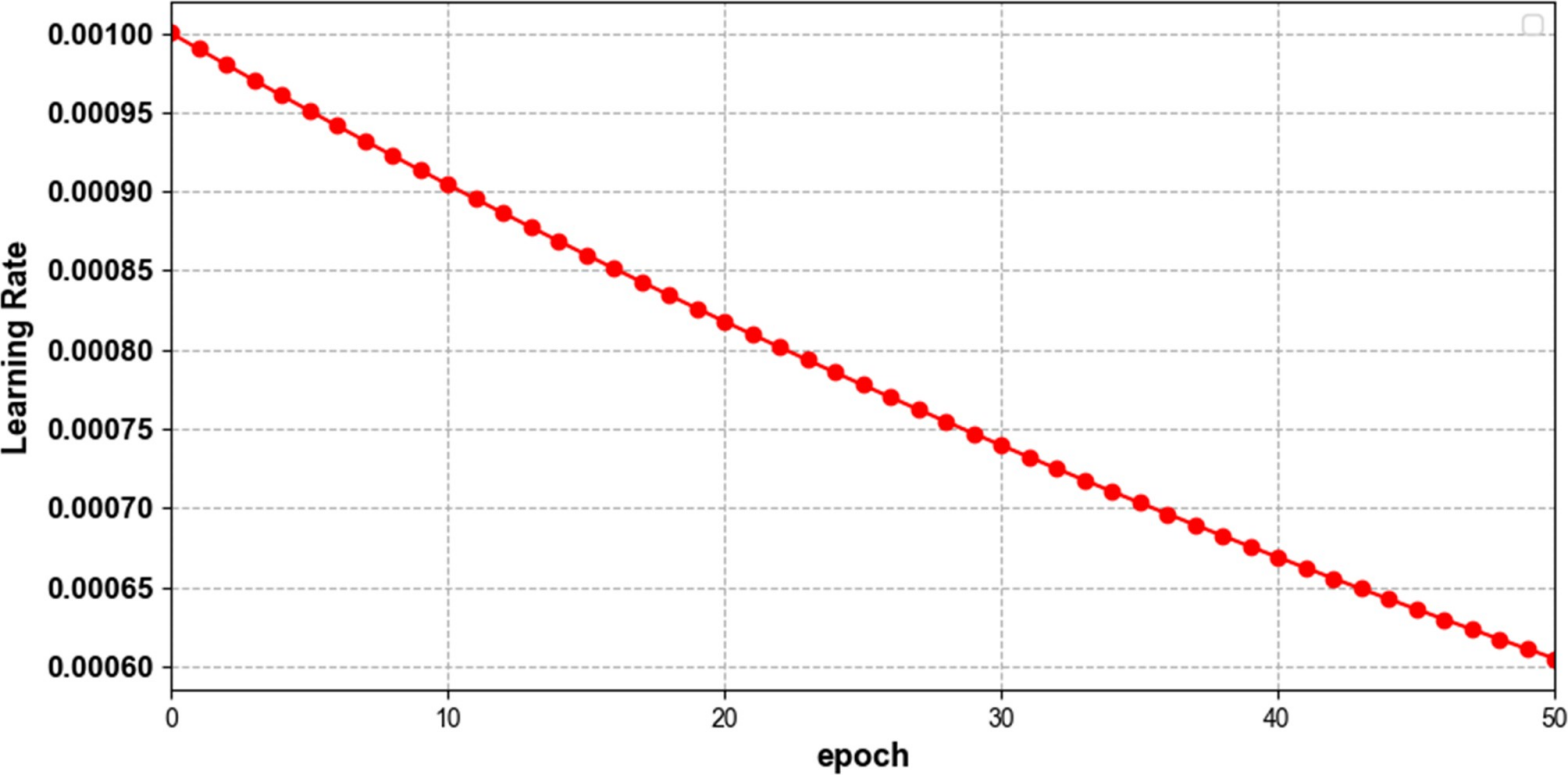
Variation of model learning rate with epoch at CR = 0.1.

The number of training rounds was 50 per round, and the training and test sets were randomly divided 0.8:0.2 using the train_test_split in Keras, with the random_state value set to 0, and the batch size is 64, the weights are updated using the Adam optimization function, and the initial learning rate is set to 0.001. In order to speed up the training and improve the model performance, an adaptive mode in which the learning rate is automatically modified with the training process is set in the network. Fig 6 shows the change of the learning rate for 50 training epoch at CR = 0.1. The learning rate decreases gradually from 0.001 at the beginning with the number of training rounds to about 0.0006 at the 50th epoch.

**Fig 7:**
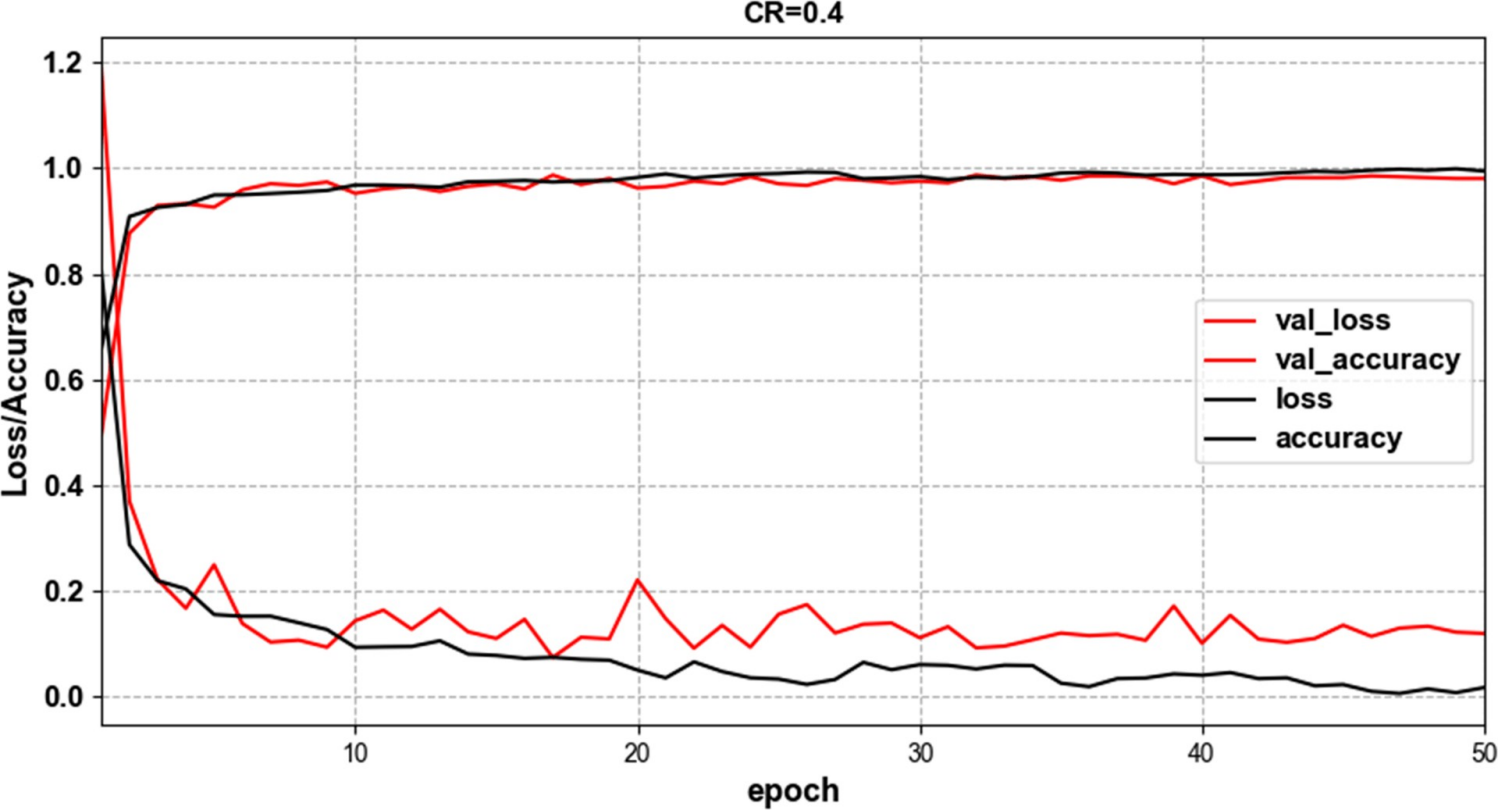
Training and validation performances using a proposed model with ECG datasets.

We have chosen the cross-entropy loss function as our loss function, which is suitable for multiclassification work, with the following equation:

$$L=\frac{1}{N}\sum_{i}L_{i}=\frac{1}{N}\sum_{i}\sum_{c=1}^{M}y_{ic}\log(p_{ic})$$ (3)

where M denotes the number of categories *y_ic_* is a symbolic function that takes 1 if the true category of sample *i* is equal to c and 0 otherwise. *p_ic_* denotes the predicted probability that the observed sample *i* belongs to category c. Fig 7 shows the changes of precision and loss value during model training when CR = 0.4, which proves that our model is convergent.

### 4.2 Evaluation metrics

In order to accurately evaluate the performance of the model, the evaluation criteria chosen in this paper are accuracy, precision, sensitivity and f1-score. Each parameter is defined as follows:

$$Accuracy=\frac{TP+TN}{TP+TN+FP+FN}$$ (4)

$$Precision=\frac{TP}{TP+FP}$$ (5)

$$Sensitivity=\frac{TP}{TP+FN}$$ (6)

$$F1=\frac{2\cdot Precision\cdot Sensitivity}{Precision+Sensitivity}$$ (7)

$$Score={\left(\frac{1}{n}\sum F1\right)}^{2}$$ (8)

where TP denotes positive samples predicted by the model as positive class, TN denotes negative samples predicted by the model as negative class, FP denotes negative samples predicted by the model as positive class, and FN denotes positive samples predicted by the model as negative class.

### 4.3 Comparison with different compression methods

We discard the traditional compression method and use a combination of three convolutional layers with a maximum pooling layer to compress the signal. In order to verify the effectiveness and advancement of the proposed compression method, we compared it with several commonly used compression algorithms while keeping the classification model unchanged. Table 4 shows the accuracy of the compression model proposed in this paper in the MIT-BIH arrhythmia database with different compression methods on six compression ratios. When CR = 0.5, the accuracy of our method on the test set is 98.82%, and when CR = 0.05, the accuracy is 97.77%. The classification accuracy of our method is higher than that of other compression methods under each compression ratio. It is proved that our proposed compressed network is more beneficial to classification than other compression methods.

**Table 4:** Accuracy of different compression methods in the MIT-BIH arrhythmia database test set at different compression ratios.

CR	compression method				
	Proposed	SVD	PCA	LEE	SAE
0.5	**98.82**	98.11	97.77	97.37	98.03
0.4	**98.55**	97.90	97.50	97.24	98.03
0.3	**98.42**	97.77	97.24	96.32	97.90
0.2	**98.16**	97.63	96.98	96.06	97.63
0.1	**98.03**	97.50	96.85	95.27	97.24
0.05	**97.77**	97.50	96.58	95.14	97.11

**Fig 8:**
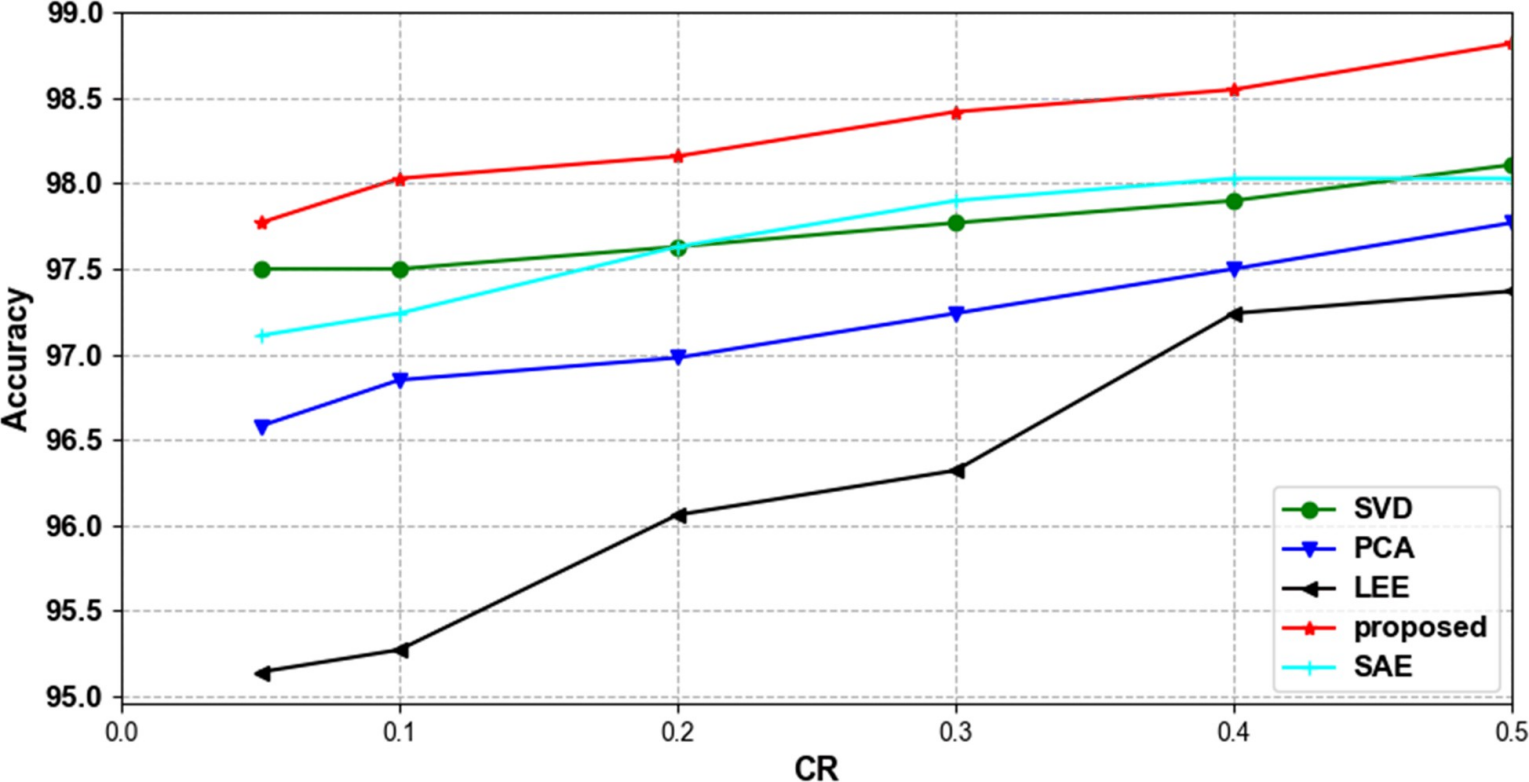
Accuracy trends in the MIT-BIH arrhythmia database using different compression methods at different compression ratios.

**Fig 9:**
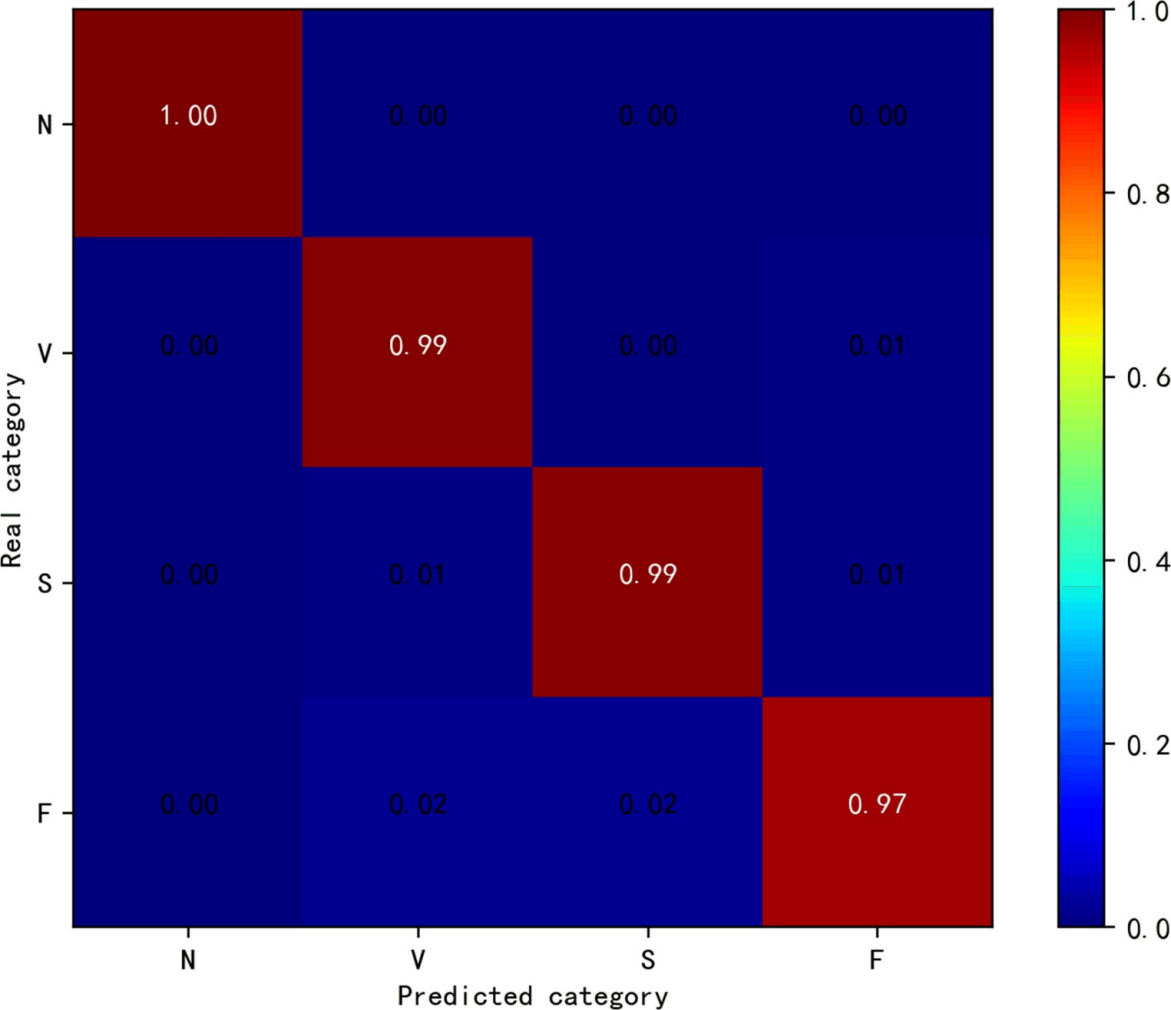
Confusion matrix of the proposed model on the MIT-BIH arrhythmia database test set at CR = 0.5.

Fig 8 shows the trend of accuracy using different compression methods with different compression ratios in the MIT-BIH arrhythmia database. All compression methods yield classification accuracies that increase with CR. It is proved that the accuracy of different compression methods increases with the increase of data volume. Fig 9 shows the confusion matrix of our proposed method for classification on the test set when CR = 0.5. Our proposed method is able to identify most compressed ECG beats with high classification ability for all four categories, N, V, S and F.

### 4.4 Comparison with different classification methods

**Fig 10:**
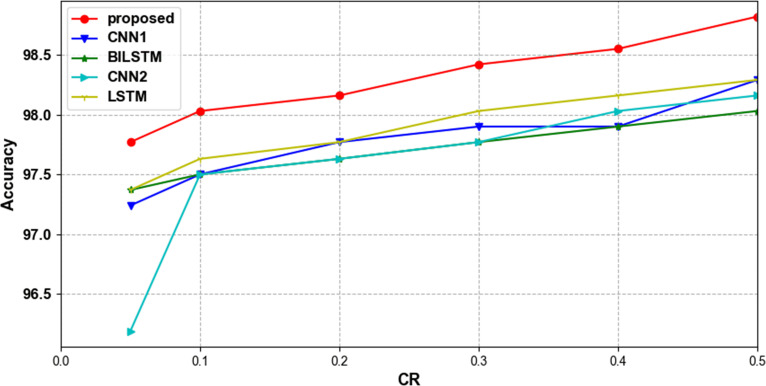
Trends in accuracy using different classification methods at different compression ratios in the MIT-BIH arrhythmia database.

**Fig 11:**
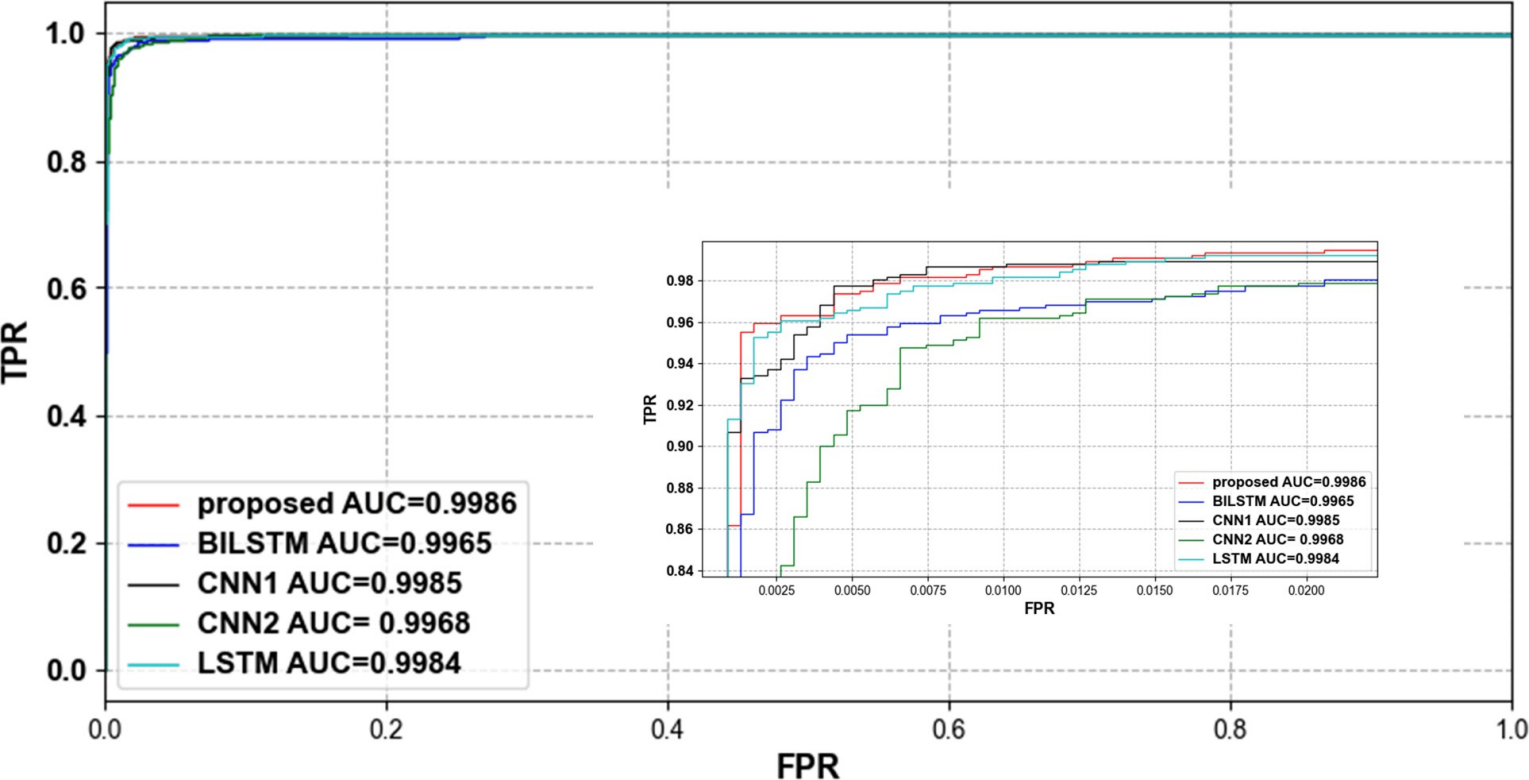
ROC curve of each classification model when CR = 0.05.

**Fig 12:**
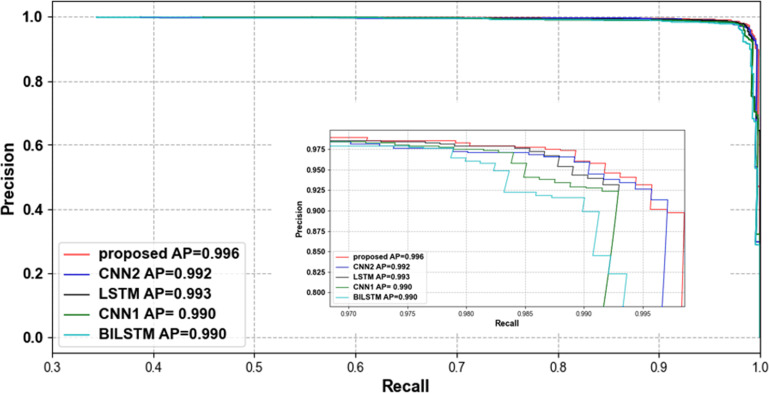
PR curve of each classification model when CR = 0.05.

To verify that our proposed network can effectively classify compressed ECG signals, we compared several lightweight networks that are also used for ECG signal classification. Table 5 shows the accuracy of different classification methods with different compression ratios. Table 6 shows the time spent in parameter training for each classification network. The parameter training time of all classification models decreases with the reduction of CR, which proves that the compressed data can be classified quickly. When CR = 0.1 and 0.05, our proposed method consumes the shortest training time, which proves that our model can do faster and better classification under the condition of very little data. Tables 7 and 8 show the average precision, sensitivity and f1-score of each network at CR = 0.4 and CR = 0.2. Our proposed classification model outperforms the other models in all metrics, with only 0.02 less sensitivity than the BILSTM model at CR = 0.4. Fig 10 shows the trend of accuracy using different classification methods with different compression ratios in the MIT-BIH arrhythmia database. Our proposed method fluctuates relatively steadily with compression ratio and has higher classification accuracy than other methods at all compression ratios. Figs 11 and 12 show the ROC curve and PR curve of each classification model when CR = 0.05.

**Table 5:** Accuracy of different classification methods in the MIT-BIH arrhythmia database test set at different compression ratios.

CR	Classification Method				
	Proposed	CNN1 [52]	BILSTM [53]	CNN2[4]	LSTM[31]
0.5	**98.82**	98.29	98.03	98.16	98.29
0.4	**98.55**	97.90	97.90	98.03	98.16
0.3	**98.42**	97.90	97.77	97.77	98.03
0.2	**98.16**	97.77	97.63	97.63	97.77
0.1	**98.03**	97.50	97.50	97.50	97.63
0.05	**97.77**	97.24	97.37	96.19	97.37

**Table 6:** The time (s) spent in parameter training for each classification network.

CR	Classification Method				
	Proposed	CNN1	BILSTM	CNN2	LSTM
0.5	90.1	89.2	174.3	**74.4**	176.2
0.4	83.8	85.4	154.4	**70.0**	160.0
0.3	76.9	77.1	129.5	**63.8**	137.7
0.2	69.6	72.2	105.9	**60.8**	99.2
0.1	**56.8**	60.3	80.2	57.7	72.0
0.05	**50.6**	55.5	71.7	52.3	59.7

**Table 7:** Average precision, sensitivity and F1-score of each classification network at CR = 0.4.

CR = 0.4	Precision	Sensitivity	F1-score
Proposed	**98.13**	98.06	**98.01**
CNN1	98.08	97.97	97.90
BILSTM	97.92	**98.08**	97.94
CNN2	97.15	97.53	97.22
LSTM	97.95	97.80	97.74

**Table 8:** Average precision, sensitivity and F1-score of each classification network at CR = 0.2.

CR = 0.2	Precision	Sensitivity	F1-score
Proposed	**98.25**	**98.09**	**98.06**
CNN1	98.21	97.86	97.85
BILSTM	97.08	96.62	97.08
CNN2	97.39	97.64	97.44
LSTM	97.72	98.06	97.83

### 4.5 Experiment on another database

To verify the robustness of the classification model, we also chose the database provided by AliCloud Tianchi for heartbeat signal classification prediction, which provides 100,000 heartbeat data, each with a length of 205 data volume, divided into a total of four categories, corresponding to the four labels 0, 1, 2, and 3 [54]. We randomly took 1000 data in each of the four categories to form a new dataset consisting of 4000 data for the experiment. The experimental parameters were set as before, again at the six compression ratios of CR = 0.5, CR = 0.4, CR = 0.3, CR = 0.2, CR = 0.1, and CR = 0.05.

**Fig 13:**
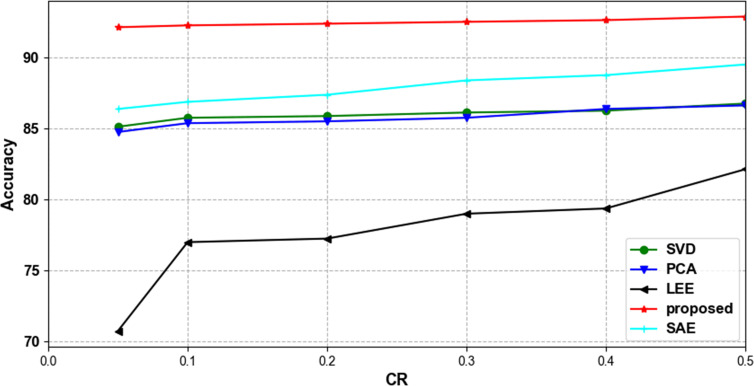
Accuracy trends using different compression methods at different compression ratios in the TianChi ECG signal database.

**Fig 14:**
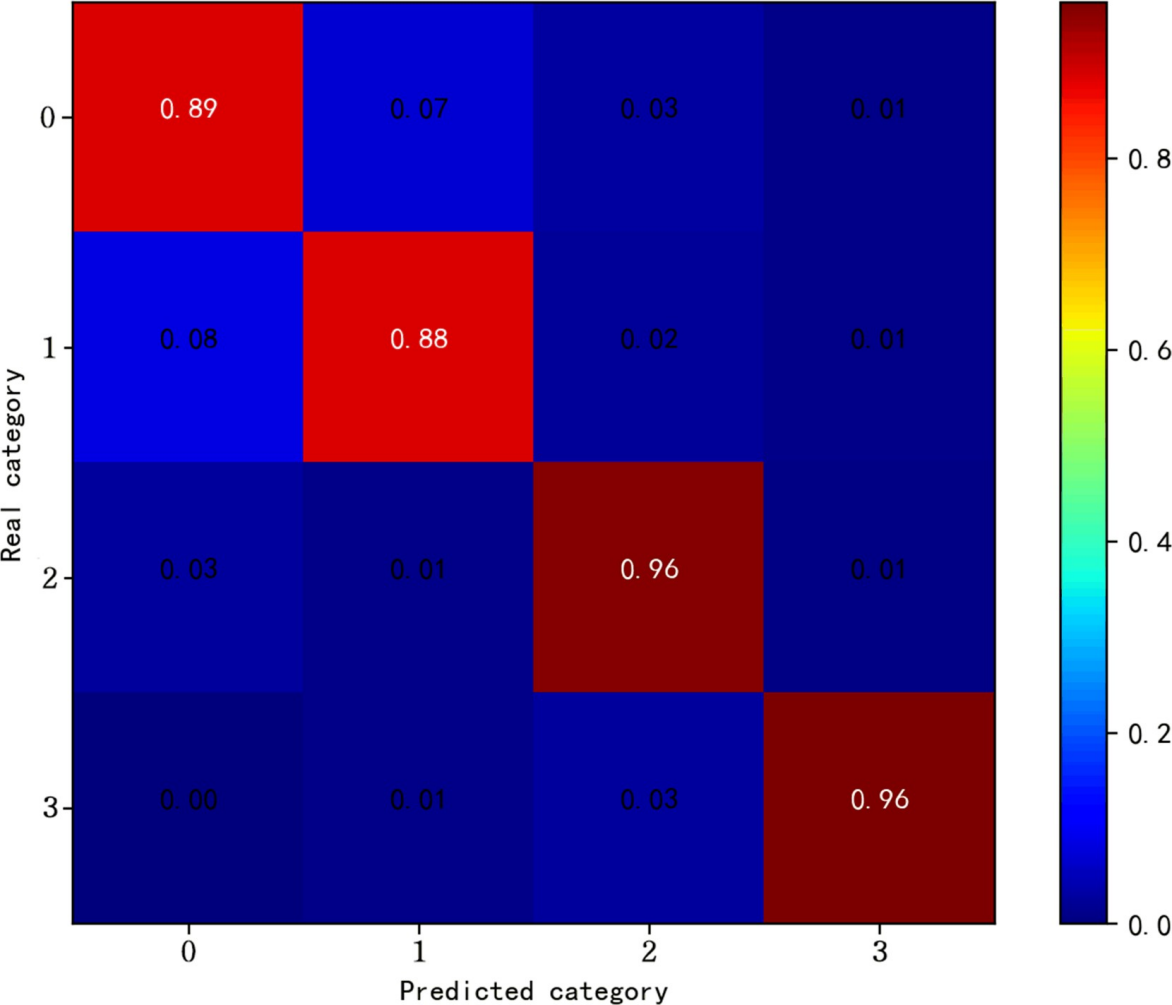
Confusion matrix of the proposed model on the TianChi ECG signal database test set at CR = 0.1.

Firstly, different compression methods are compared, and the classification method proposed in this paper is chosen to keep the same, and the experimental results obtained by classification under different compression ratios are shown in Table 9. Our proposed method has 92.87% accuracy in this database at CR = 0.5, which is a 3.37% improvement compared to the closest SAE of 89.50%. Fig 13 shows the trend of accuracy at different compression ratios. It can be seen that the method proposed in this paper is ahead of other compression methods at all compression ratios and is the most stable, with only 0.75% difference between the maximum and minimum values. Fig 14 shows the confusion matrix of our proposed method for classification on the test set at CR = 0.1. From the confusion matrix, we can see that our proposed method is generally effective in classifying heartbeat fragments with labels 0 and 1, but has a higher recognition accuracy for heartbeat fragments with labels 2 and 3. The reason for this may be because the features of class 0 and class 1 heartbeat fragments in this database are relatively similar, resulting in some labels misidentified each other.

**Table 9:** Accuracy of different compression methods in TianChi ECG signal database test set at different compression ratios.

CR	compression method				
	Proposed	SVD	PCA	LEE	SAE
0.5	**92.87**	86.75	86.62	82.13	89.50
0.4	**92.62**	86.25	86.37	79.37	88.75
0.3	**92.50**	86.12	85.75	79.00	88.38
0.2	**92.37**	85.87	85.50	77.25	87.37
0.1	**92.25**	85.75	85.37	77.00	86.87
0.05	**92.12**	85.12	84.75	70.75	86.37

**Fig 15:**
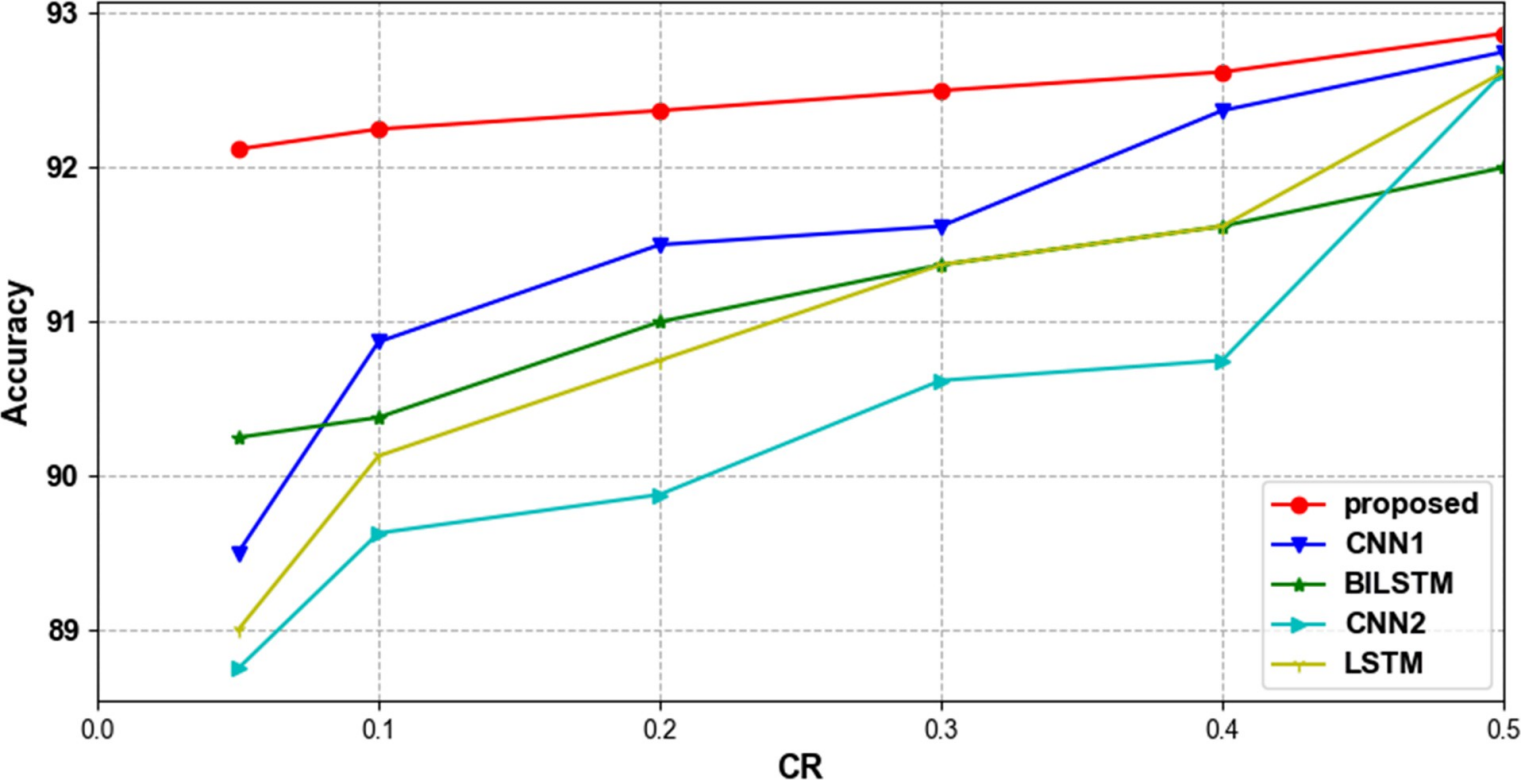
Accuracy trends using different classification methods at different compression ratios in the TianChi ECG signal database.

Then the compression method proposed in this paper is chosen to keep the same, and the different classification methods are compared. Table 10 shows the experimental results obtained from the classification under different compression ratios. Fig 15 shows the accuracy trends using different classification methods at different compression ratios. The classification method proposed in this paper also outperforms other classification methods in terms of accuracy and is more stable for classifying data with different compression ratios under this database. Combined with the experimental results, we can see that under the new data set, the proposed method can still effectively compress and classify ECG signals, and is superior to the same type of compression and classification methods.

**Table 10:** Accuracy of different classification methods in the TianChi ECG signal database test set at different compression ratios.

CR	Classification Method				
	Proposed	CNN1 [52]	BILSTM [53]	CNN2 [4]	LSTM [31]
0.5	**92.87**	92.75	92.00	92.62	92.62
0.4	**92.62**	92.37	91.62	90.75	91.62
0.3	**92.50**	91.62	91.37	90.62	91.37
0.2	**92.37**	91.50	91.00	89.88	90.75
0.1	**92.25**	90.87	90.38	89.63	90.13
0.05	**92.12**	89.50	90.25	88.75	89.00

### 4.6 Ablation experiment

**Fig 16:**
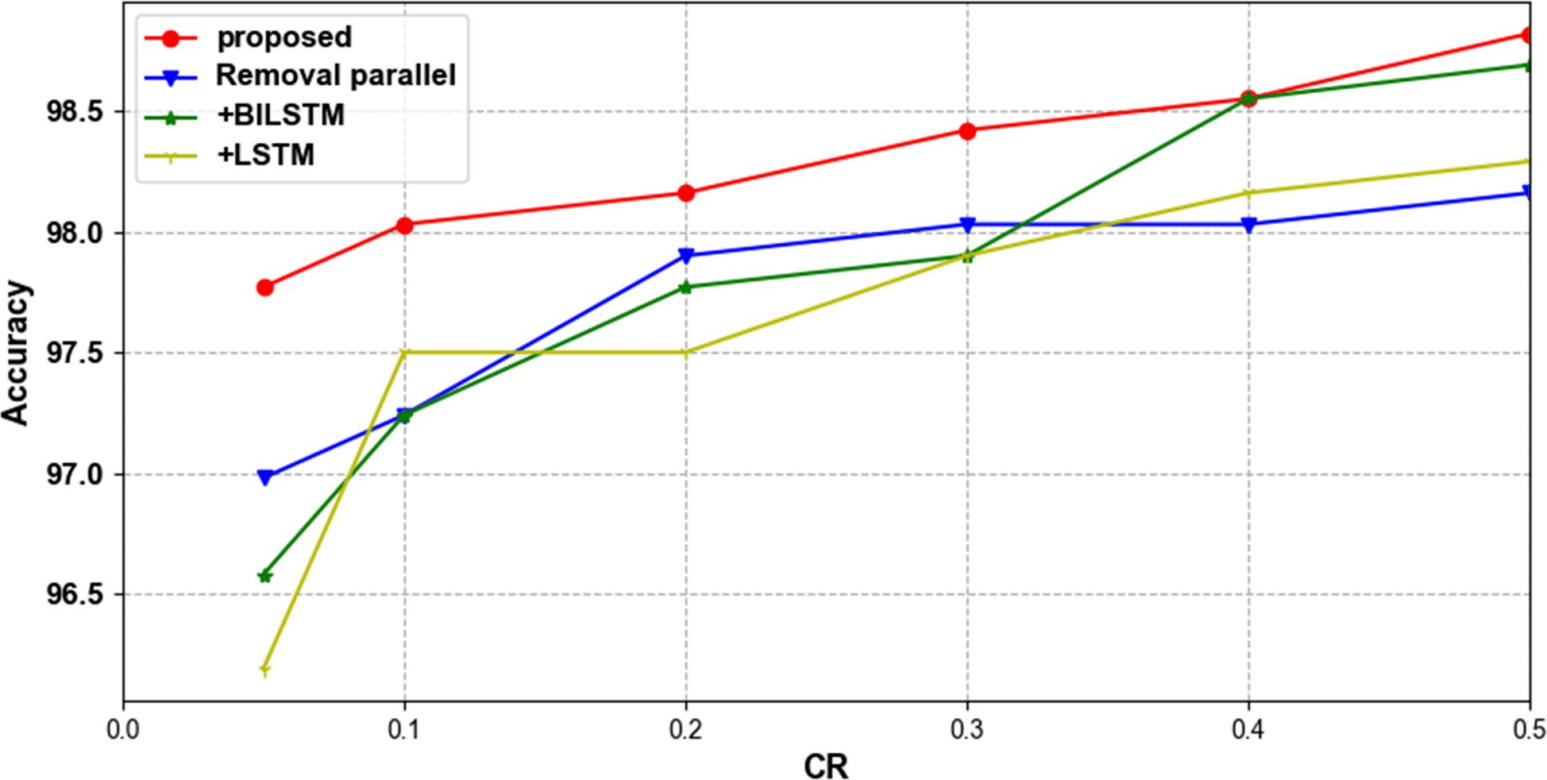
Accuracy trends using different classification methods at different compression ratios in the MIT-BIH ECG signal database.

To verify the validity of the proposed classification model, two ablation experiments were performed on the MIT-BIH ECG signal database. The first set was to delete the parallel convolution structure and directly use three convolution blocks for classification. The second group is to add LSTM or BILSTM layer after three convolution blocks. Table 11 and Fig 16 shows the classification results of ablation experiments under different compression ratios. After deleting the parallel convolution structure or adding LSTM and BILSTM layers, the accuracy of the obtained classification network is inferior to the original one. Ablation experiments show that although LSTM and BILSTM are often used to classify sequential signals, they are not as effective as the convolutional layer in classifying compressed ECG signals.

**Table 11:** Accuracy of different classification methods in the MIT-BIH ECG signal database test set at different compression ratios.

CR	Classification Method			
	Proposed	Removal parallel	+LSTM	+BILSTM
0.5	**98.82**	98.16	98.29	98.69
0.4	**98.55**	98.03	98.16	98.55
0.3	**98.42**	98.03	97.90	97.90
0.2	**98.16**	97.90	97.50	97.77
0.1	**98.03**	97.24	97.50	97.24
0.05	**97.77**	96.98	96.19	96.58

## 5. Conclusion

We propose a classification model for identifying compressed ECG signals. In order to save the energy consumption of wearable devices, we compress the lengthy ECG signals before classification, which **not** only reduces the storage space but also increases the classification speed and ensures the classification accuracy. The experimental results show that both the compression and classification methods included in our proposed model outperform other traditional methods and are well suited to the task of classification of compressed signals.
